# Supplementary material for: A Dual and Rapid RPA-CRISPR/Cas12a Method for Simultaneous Detection of Cattle and Soybean-Derived Adulteration in Goat Milk Powder
Source: Foods. 2024 May 24;13(11):1637. doi: 10.3390/foods13111637 (PMC11172236; doi:10.3390/foods13111637)
Supplement: Supplementary file 1 [file foods-13-01637-s001.zip › foods-2983607-supplementary.pdf]

Supplementary File

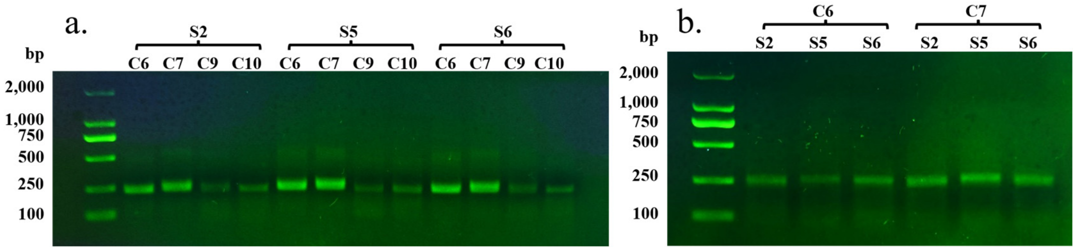

Figure S1: Amplification result based on the RPA primers. (a) Cattle genomic DNA. (b) Soybean genomic DNA. NTC: nontarget control.

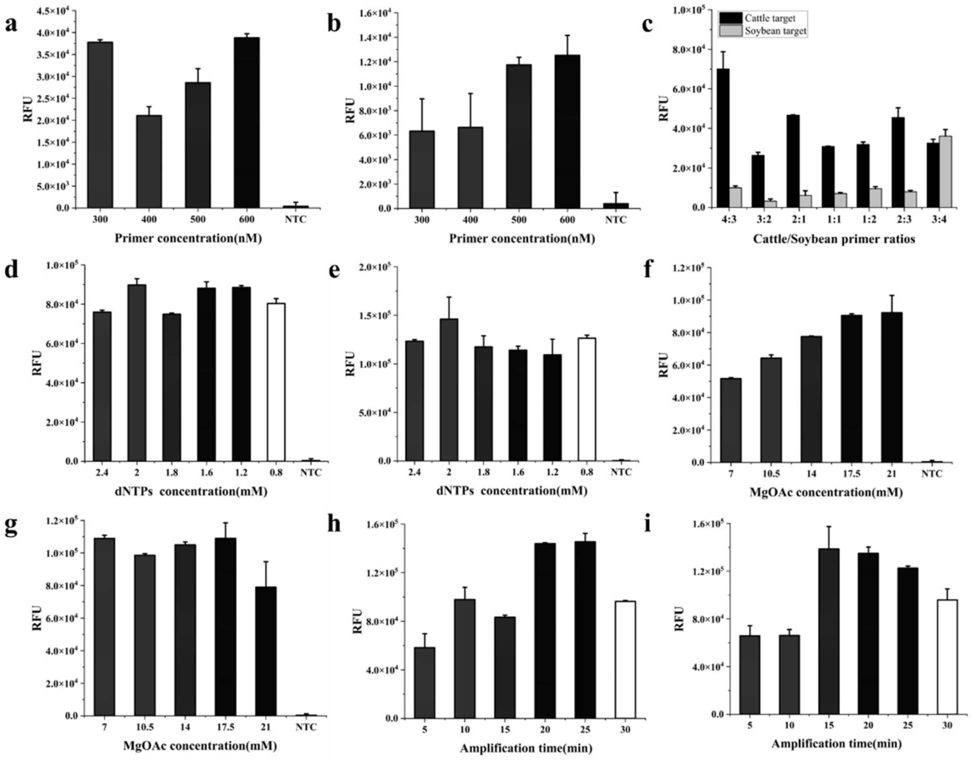

Figure S2: Condition optimization of the dual RPA amplification system. (a) Cattle Primer concentration. (b) Soybean Primer concentration. (c) Primer ratios for cattle and soybean. (d) dNTPs concentration in the cattle DNA amplification. (e) dNTPs in the soybean DNA amplification. (f) MgOAc concentration in the cattle DNA amplification. (g) MgOAc concentration in the soybean DNA amplification. (h) Amplification time of the cattle DNA. (i) Amplification time of the soybean DNA. NTC: nontarget control.
